# Image Calibration in choroidal vascularity index measurement

**DOI:** 10.1186/s40942-023-00508-2

**Published:** 2023-11-10

**Authors:** Kiana Hassanpour, Hamid Ahmadieh

**Affiliations:** Ophthalmic Research Center, Research Institute for Ophthalmology and Vision Science, Shahid Beheshti University of Medical Sciences, Tehran, Iran

Dear Editor

We thank Dr. Motamed Shariati for his interest in our article titled “choroidal structure investigated by choroidal vascularity index in patients with inherited retinal disease” [1].

In calculating the choroidal vascularity index (CVI), we used the method proposed by Sonada et al. [2], fully described step by step in the supplementary material attached to this response. In the last picture of the supplementary method, there is a detailed picture depicting the importance of the use of “set scale” in the calculation process (Fig. 1).

**Fig. 1:**
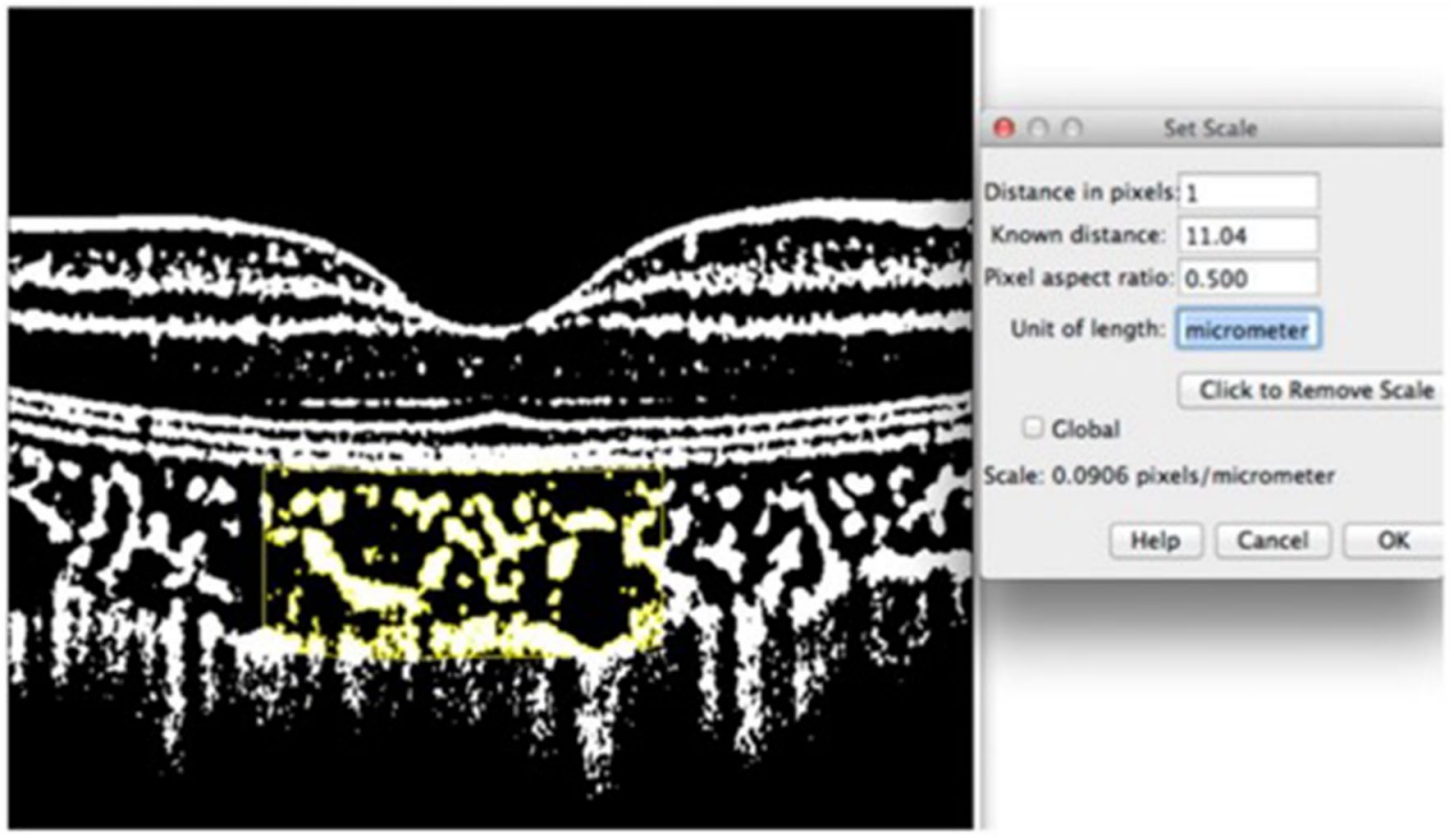
The supplementary material describes the Sonada’s method for calculating the choroidal vascularity index (CVI)

At the “analyze” section of the software toolbar in the “set scale” window, there is an option named “pixel aspect ratio”. The proportion of width to height for subsequent measurements can be set to any value applying this option. As shown in Fig. 1, the “pixel aspect ratio” is written 0.5 to compensate for the horizontal to vertical measure. In order to implement this technique, we used the “straight” tool to draw two straight lines at both horizontal and vertical dimensions at the scale bar, separately. A suggested value for the length of each drawn line would appear in the toolbar and then the ratio of the width to the length could be calculated according to these suggested values. In the next step, the calculated value is employed to fill the “pixel aspect ratio” box. Regarding the scale demonstrated on the left side of the image, this ratio was 0.3 in our images. This means the measurement could be done solely by adjusting the horizontal dimension in the scale bar below the image and the scale of height measurements of the image would be computed by software.

It should also be mentioned that in order to draw a perfectly straight line and avoid a crooked line, the shift button in the keyboard was used to omit any possible mistake in the process of calibrating the values. Therefore, this step of calculation of CVI would compensate for the concern raised by Dr. Motamed Shariati.

Regarding the larger area of TCA, we agree with the authors. Sonada et al. [2] selected an area with a total width of 1500 microns [2]. However, there is a controversy in this regard and some authors have chosen larger areas for further declaration of the possible differences [3, 4]. In the present study, we selected a region of interest with a total width of 3000 microns; thus, this could explain the larger area of TCA reported in our study.

## Data Availability

The datasets used and/or analyzed during the current study are available from the corresponding author on reasonable request.
